# Molecular characterization and genetic relatedness of clinically *Acinetobacter baumanii* isolates conferring increased resistance to the first and second generations of tetracyclines in Iran

**DOI:** 10.1186/s12941-017-0226-9

**Published:** 2017-07-19

**Authors:** Zahra Meshkat, Himen Salimizand, Yousef Amini, Mostafa Khakshoor, Davoud Mansouri, Hadi Farsiani, Kiarash Ghazvini, Adel Najafi

**Affiliations:** 1 Antimicrobial Resistance Research Center, Bu Ali Research Institute, Mashhad, Iran; 2 Department of Microbiology and Virology, Ghaem hospital, Mashhad University of Medical Sciences, Ahmadabad Boulevard, Mashhad, Khorasan Razavi PO Box: 91766-99199 (155), Iran; 3 Department of Microbiology, Faculty of Medicine, Kurdistan University of Medical Sciences, Sanandaj, Iran; 4 Department of Microbiology, Faculty of Medicine, Zahedan University of Medical Sciences, Zahedan, Iran; 5 Microbiology Department, Islamic Azad University, Tehran, Iran; 6 Student Research Committee, Faculty of Medicine, Mashhad University of Medical Sciences, Mashhad, Iran

**Keywords:** *Acinetobacter baumannii*, Tetracyclines, Molecular epidemiology, rep-PCR, IC clone

## Abstract

**Background:**

The increasing resistance of *Acinetobacter baumannii* to antibiotics has recently been regarded as a notable therapeutic difficulty. Evaluating resistance rates of some *A. baumannii* isolates to tetracyclines had an impact on understanding the antibiotic resistance dissemination. By comparing genetic characteristics and relatedness of *A. baumannii* isolates, we are able to determine the transition dynamics of outbreak isolates.

**Methods:**

A total of 72 non-duplicate isolates of *A. baumannii* were recovered in 2011 and 2015 and minimum inhibitory concentration (MIC) range distribution of the isolates to tetracyclines was performed by broth micro dilution (BMD) assay, and to determine the lineage relatedness of the outbreak isolates repetitive extragenic palindromic element based on polymerase chain reaction (rep-PCR) and international clonal (ICs) investigations were performed.

**Results:**

Resistance rates to tetracycline, doxycycline and minocycline in 2011 were 73, 2 and 0%, while these rates in 2015 increased up to 90, 84 and 52%, respectively. The *tetB* existed in 100% of all the isolates of both years. *tetA* was not found in any of the isolates. According to the rep-PCR assays, up to 83% of all isolates clustered distinctly and only 6% of isolates had a common root. The percentage rates of IC1 decreased from 42% in 2011 to 22% in 2015, while those of IC2 increased from 28 to 36%, from 2011 to 2015.

**Conclusions:**

Our data showed that resistance to the first and second generations of tetracyclines is on the rise and the clonal transition dynamics of isolates are in progress in our hospital.

## Background

*Acinetobacter baumannii* as an opportunistic pathogen has recently been known as a nosocomial pathogen which is associated with health care infections [1]. Due to the increasing antimicrobial resistance, *A. baumannii* has emerged as a life-threatening pathogen in the three past decades. The most important mechanisms of resistance to tetracyclines in *A. baumannii* isolates are efflux pumps followed by ribosomal protections and enzymatic inactivation. To date, tetracyclines were considered as a second-line therapy for *Acinetobacter* infections. However, due to the lack of any new under-development antibiotics and decreased effectiveness of the first-line antibiotics, older ones (e.g. tetracyclines) have been taken into consideration again [2]. Streptomyces species were the source of tetracycline, which is considered as the first generation of tetracyclines. Semisynthetic tetracyclines, doxycycline and minocycline, as the second generation of this group have wider spectrum. Tigecycline, a glycylcycline, has recently approved by US Food and Drug Administration (FDA) antibacterial agent. This minocycline structural analogue is indicated intravenously for the treatment of complicated infections [3].

Resistance to the first and second generations of tetracyclines in *A. baumannii* isolates mainly resulted from the acquired major facilitator superfamily (MFS) efflux pumps, including *tetA*, *tetB*, *tetG*, *tetH*, *tetL*, and *tet39,* resistance nodulation division family (RND) efflux pumps nominated as *adeABC*, *adeIJK*, *adeFGH*, *adeM*, *adeDE* [4], and finally ribosomal protections and enzymatic inactivation [5]. *tetA* is responsible for the resistance to tetracycline and doxycycline whereas *tetB* has been found in the isolates that were also resistant to minocycline [6]. Notably, the main resistance mechanisms of tetracyclines are *ade* efflux systems while coexistence with *tetA* and *tetB* determinants resulted in increasing MIC values [7].

Due to the lack of accuracy in disc diffusion method, determining susceptibility to doxycycline and minocycline was recommended to be carried out by Epsilometric test (E-test) or broth microdilution assays [8].

Regarding the impact of *A. baumannii* in healthcare—associated infections and rapid spreading of antibiotic-resistant strains, epidemiological investigation and determination of the clonal relationships among *A. baumannii* isolates should be considered. Some molecular techniques are currently at our disposal for typing and to clonal relatedness among clinically isolates of *A. baumannii*. The most common assay is the repetitive extragenic palindromic polymerase chain reaction-based (rep-PCR) that is simple, rapid and reliable compared to PFGE. Determining international clonal lineages (ICs) in *A. baumanii* is a useful tool for showing widespread distribution of global distributed clones [9]. Outbreak strains which are more resistant to antibiotics and might be associated with specific clinical syndromes, could be identified by such aforementioned studies [10]. The present study aimed to compare the resistance range of tetracycline, doxycycline and minocycline in the two periods of 2011 and 2015. Moreover, resistance determinants *tetA* and *tetB* were screened in clinical isolates of *A. baumannii*. Also, to determine the clonal transition dynamics and relatedness of isolates, international clonal lineage investigation and rep-PCR assays were performed in Ghaem Hospital, Mashhad, Iran.

## Methods

### Bacterial isolates and hospital setting

A total of 72 non-duplicate isolates of carbapenem resistant *A. baumannii* (CRAB) were collected from admitted patients at the Intensive Care Units (ICUs) of Ghaem Hospital (1000-bed referral university hospital, with NICU, PICU and adult’s ICU wards). The ethics committee of hospital and institutional review boards approved this study, and recommendations by STROBE were considered to report the results [11]. Thirty-six isolates were collected in 2011, and the other 36 isolates were obtained in 2015. In the first stage, these isolates were identified by API20NE as *Acinetobacter baumannii*-*calcoaceticus* complex, and for confirming as *A. baumannii*, all of the isolates were investigated by OXA-51-like β-lactamase and *gyrB* multiplex PCR amplification according to previous studies [12]. The isolates were stored in 30% v/v glycerol/triptic soy broth medium at −70 °C.

### Antibacterial susceptibility testing and efflux pump activity evaluation

In order to evaluate antimicrobial susceptibility to doxycycline, minocycline and tetracycline, broth micro dilution method was used according to CLSI M07-A9 instructions [13] and the results were interpreted according to CLSI M100-S25 guidelines [14]. In this study, the intermediate isolates were also considered as resistant. All antibiotic chemicals were purchased from Sigma Chemicals Co., Inc. (St. Louis, USA). *Escherichia coli* ATCC 25922 and *Pseudomonas aeruginosa* ATCC 27853 were used as reference strains.

For tetracyclines resistant isolates, MICs of the three aforementioned antibacterials were repeated in the presence of the following efflux pump inhibitors (EPIs) by broth microdilution method. Carbonyl cyanide 3-chlorophenylhydrazone (CCCP), phenyl-arginine-β-naphthylamide (PAβN), 1-(1-naphthylmethyl)-piperazine (NMP), reserpine and verapamil (Sigma), CCCP, PAβN, NMP, reserpine, and verapamil were added to the broth at the final concentrations of 5, 70, 100, 50, and 100 g/L, respectively [4]. A fourfold or greater decrease in the MIC values in the presence of EPIs was considered as significant inhibition.

### PCR amplification of *tetA and tetB*

The existence of tetracycline resistant determinants, *tetA* and *tetB,* were detected by the PCR assay according to previous studies with some modifications [15]. Briefly, the template DNA for PCR reactions was obtained from heating 200 μL suspension of overnight bacterial cultures for 10 min at 95 °C and quickly refrigerating for 10 min followed by centrifugation at 15,000*g* for 5 min, and then discarding the supernatant. PCR products were analyzed by electrophoresis on agarose gels 2% using a 100plus DNA ladder Fermentase (Thermo Fisher Scientific, Germany) as a size marker.

### Clonal and international lineage relatedness

To discover clonal relationships among the isolates of *A. baumannii*, we exploited two molecular typing methods. All the isolates were subjected to rep-PCR and international clonal lineage (IC) investigations.

A pair of primers with indication for REP sequences of *A. baumannii* were used as amplification primers, as described previously. The concentration of REP primers, REP1 and REP2, were 50 pmol/mL [16]. PCR products were detected by electrophoresis on 2% agarose using a 100 bp plus DNA ladder (Thermo Fisher Scientific, Germany), as a size marker. GelJ software (v. 1.3) was utilized in order to analyze the band patterns, considering dice tolerance 2.0 and UPGMA method to depict dendrogram. Isolates with at least 95% similarity were considered related and defined as the same rep-PCR cluster. IC lineages detection was done by a multiplex PCR assay of all the strains to find *csuE*, OXA-66/69 and *ompA* genes [9].

### Statistical study

All statistical analyses were performed using SPSS v.16.0. A univariate analysis was carried out using Pearson’s Chi square test for nominal variables in two independent groups. A P value of <0.05 was considered significant.

## Results

### Antimicrobial susceptibility and molecular detection of *tetA and tetB*

In two periods of 2011 and 2015, 72 CRAB isolates (36 isolates per year) were collected. The percentages of tetracyclines resistant isolates in 2015 were obviously higher than those in 2011. About 70% (26 of 36) of isolates in 2011 were resistant to tetracycline, while in 2015, the resistance rates were increased up to 90% (32 of 36). The increasing rates were also observed for doxycycline and minocycline. In 2011, 2.7% (1 of 36) of the isolates were resistant to doxycycline versus 84.2% (30 of 36) in 2015. None of the isolates were resistant to minocycline in 2011, but the resistance rate increased to 52.6% (19 of 36) after 4 years (Table 1).

**Table 1:** Broth microdilution susceptibilities and MIC range distribution of 72 *A. baumannii* isolates (results represented by %)

	2011			2015		
Agent/MIC_mg/L_	≤4	8	≥16	≤4	8	≥16
TET	27	33	40	10	2.6	87.4
DOX	98	0	2	15.8	0	84.2
MIN	100	0	0	47.4	31.6	21

*TET* tetracycline, *DOX* doxycycline, *MIN* minocycline

**Fig. 1:**
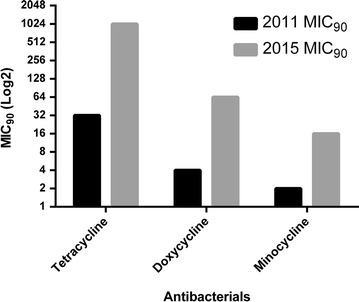
MIC_90_ comparison of tetracycline, minocycline and doxycycline in 36 isolates in 2011 versus 36 isolates in 2015

MIC_90_ of minocycline, doxycycline and tetracycline were dramatically increased after passing 4 years (Fig. 1). MICs_90_ for minocycline, doxycycline and tetracycline in 2011 were 2, 4 and 32 mg/L, respectively, whereas in 2015 they obviously increased to 16, 64 and 1024 mg/L, respectively. The MICs of resistant isolate for these antibacterials in the presence of EPIs were significantly changed. About 88% of tetracycline resistant isolates (51 out of 58) showed decreasing MIC when tetracycline was presented with EPIs. The effect of EPIs on the MICs of doxycycline and minocycline resistant isolates (31 and 19 out of 72, respectively), was reduced in 26% (8 of 31) and 47% (9 of 19) of doxycycline and minocycline, respectively. The tetracycline resistance determinant *tetB* was detected in all of the isolates of both years, however, tetA was not found in any of the isolates.

### Epidemiological investigations and international clonal lineages

**Fig. 2:**
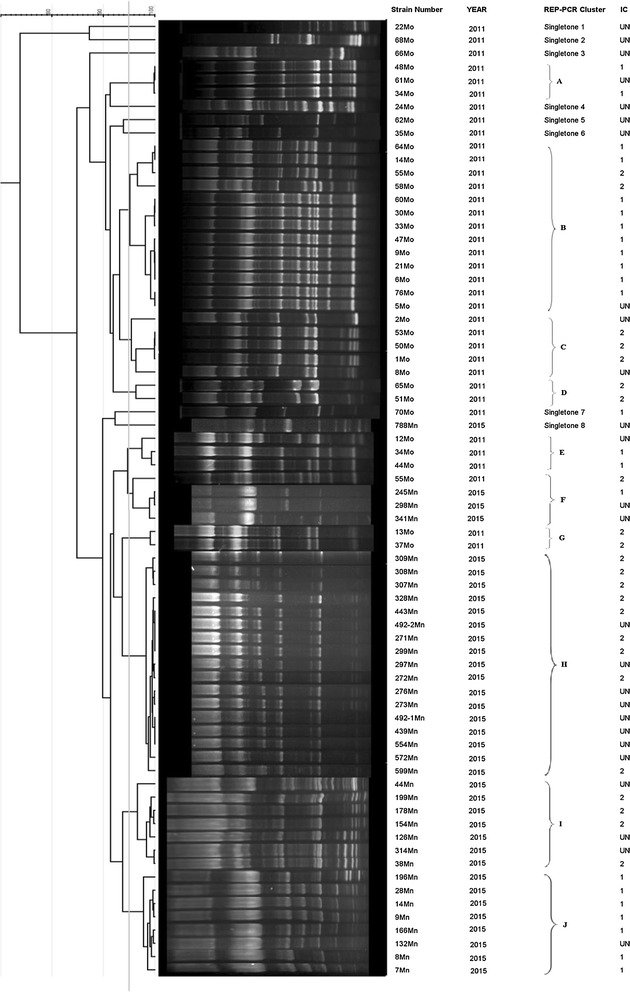
REP generated dendrogram of all isolates with considering more than 95% of similarity for clustering of isolates. *MN* Mashhad new, *Mo* Mashhad old, *UN* unknown

About 10 distinctive rep-PCR clusters (named A to J) and eight singleton isolates were inferred from the band patterns (Fig. 2). Clusters A–E and G consisted of representatives of strains of 2011, and clusters H, I and J indicated strains of 2015. Cluster F consisted of strains of both years of 2011 and 2015. Our data showed that up to 94% of all the strains were included in nine distinct clusters and only 6% of them had common roots.

Results from Multiplex PCR revealed that 42% of strains in 2011 were IC1, 28% were IC2 and 30% were unknown, while only 22% of strains in 2015 were attributed to IC1, 36% to IC2 and 42% were unknown.

## Discussion

This integrative study provides concerns about a formidable increase in drug resistance to *A. baumannii* concomitant with prospects to clonal transmission dynamics of outbreak isolates over a 4-year interval between 2011 and 2015.

Due to the increase in resistance to majority of antibiotics in *A. baumannii*, evaluating antimicrobial susceptibility of older agents that was not used in clinical practice is of interest to overcome the Acinetobacter infections. A previous study showed the satisfying effectiveness of tetracyclines against *A. baumannii* in vitro [15]. In this regard, our data showed that the resistance rates for tetracycline, doxycycline and minocycline in 2011 were 73, 2 and 0%, respectively. Meanwhile, these resistance rates increased to 90, 84.2 and 52.6% in the case of the noted antibiotics in 2015. Because of not using tetracyclines in clinical practice of *A. baumannii* infections in our hospitalized patients, the increased resistance to these three agents was not expected. Significant increasing resistance in our hospital setting is in correlation with another previous study in Iran [17]. Being consistent with our study, Maleki et al. reported that resistance rates of tetracycline, minocycline and doxycycline to *A. baumannii* isolates in Tehran in 2013 were 89, 35 and 25%, respectively.

The *tetA* and *tetB* determinants are common in tetracyclines resistant *A. baumannii* isolates [6]. Our results are also in agreement with a previous study by Farsiani et al. [18] in 2015 who reported that the *tetA* gene was not found in any of the strains whereas *tetB* was detected in 100% of the strains. In our study, the increased level of resistance to tetracyclines was observed in spite of any detectable differences in prevalence of *tetA* and *tetB* in all of the isolates. The effect of EPIs herein on most of the tetracycline resistant strains can explain that the increasing resistance is related to efflux pumps activity.

By using molecular typing methods, we are able to discriminates widespread clonal lineages of *A. baumannii* responsible for hospital clonal transition dynamics in outbreaks worldwide [19]. Amongst ten clusters obtained from evaluating all of the strains by rep-PCR, six clusters (A–E and G) included representatives of strains of 2011. The 2015 strains were classified in three clusters (H, I and J), and one cluster (F) contained strains of both years. At a glance, it can be inferred that more than 94% of all the strains were classified in distinct clusters and less than 6% of the strains are in a common (F) cluster. Furthermore, up to 91% of the strains in 2015 were classified in three clusters. Considering the decrease in the number of clusters from 2011 to 2015, it certainly indicated that new lineages with different resistance patterns were replaced over 4 years.

In this study, the percentage rates of IC1 and IC2 in the strains of 2011 were 42 and 28%, whereas in the strains of 2015 they were 22 and 36%, respectively. Higher prevalence of IC2 strains in 2015 compared with 2011, is consistent with the previous reports which state that in the recent years IC2 was a more common lineage in antibiotic-rich environments of hospitals [20]. A pervious study by Turton et al. [9] showed that IC1 strains have been prevalent in the past years. Therefore, it can be concluded that the clonal transition dynamics of strains in our hospital is in progress. The increased prevalence of IC2 in our hospital concomitant with elevated resistance to the first and second generations of tetracycline strengthens the fact that the IC 2 strains have higher resistance genetic determinants than IC1 strains. This consequence is in accordance with the previous study where IC 2 strains were relatively older than the other ICs which have been undergoing extensive diversification [21].

## Conclusions

With the best of our knowledge, this study is the first comparison of clinical strains of CRAB strains in the same location in two periods in Iran. Our data indicates that despite not using tetracyclines in treatment of Acinetobacter infections, the increased resistance to tetracyclines is likely duo to the clonal transition dynamics of strains with replacement of IC2 clones which contain higher prevalence of resistance determinants. Molecular detection of tetracycline resistance determinants in our hospital is due to the other efflux pumps rather than tetA and tetB efflux pumps. Eventually, to overcome the increasing resistance to antibiotics, some prerequisites should be considered e.g., improving antibiotic stewardship programs (ASPs), hygienic surveillance programs, epidemiological study of outbreak strains, and concrete infection control.
